# The impact of heme oxygenase-2 on pharmacological research: A bibliometric analysis and beyond

**DOI:** 10.3389/fphar.2023.1156333

**Published:** 2023-04-19

**Authors:** Cesare Mancuso

**Affiliations:** ^1^ Fondazione Policlinico Universitario A. Gemelli IRCCS, Rome, Italy; ^2^ Department of Healthcare Surveillance and Bioethics, Section of Pharmacology, Università Cattolica Del Sacro Cuore, Rome, Italy

**Keywords:** bilirubin, carbon monoxide, drug research and development, gender medicine, neuroprotection, Parkinson’s disease, translational pharmacology

## Abstract

Heme oxygenase (HO-2) is an enzyme mainly involved in the physiologic turnover of heme and intracellular gas sensing, and it is very abundant in the brain, testes, kidneys and vessels. Since 1990, when HO-2 was discovered, the scientific community has underestimated the role of this protein in health and disease, as attested by the small amount of articles published and citations received. One of the reason that have contributed to the lack of interest in HO-2 was the difficulty in upregulating or inhibiting this enzyme. However, over the last 10 years, novel HO-2 agonists and antagonists have been synthesized, and the availability of these pharmacological tools should increase the appeal of HO-2 as drug target. In particular, these agonists and antagonists could help explain some controversial aspects, such as the neuroprotective *versus* neurotoxic roles of HO-2 in cerebrovascular diseases. Furthermore, the discovery of HO-2 genetic variants and their involvement in Parkinson’s disease, in particular in males, opens new avenues for pharmacogenetic studies in gender medicine.

## 1 Introduction

**FIGURE 1:**
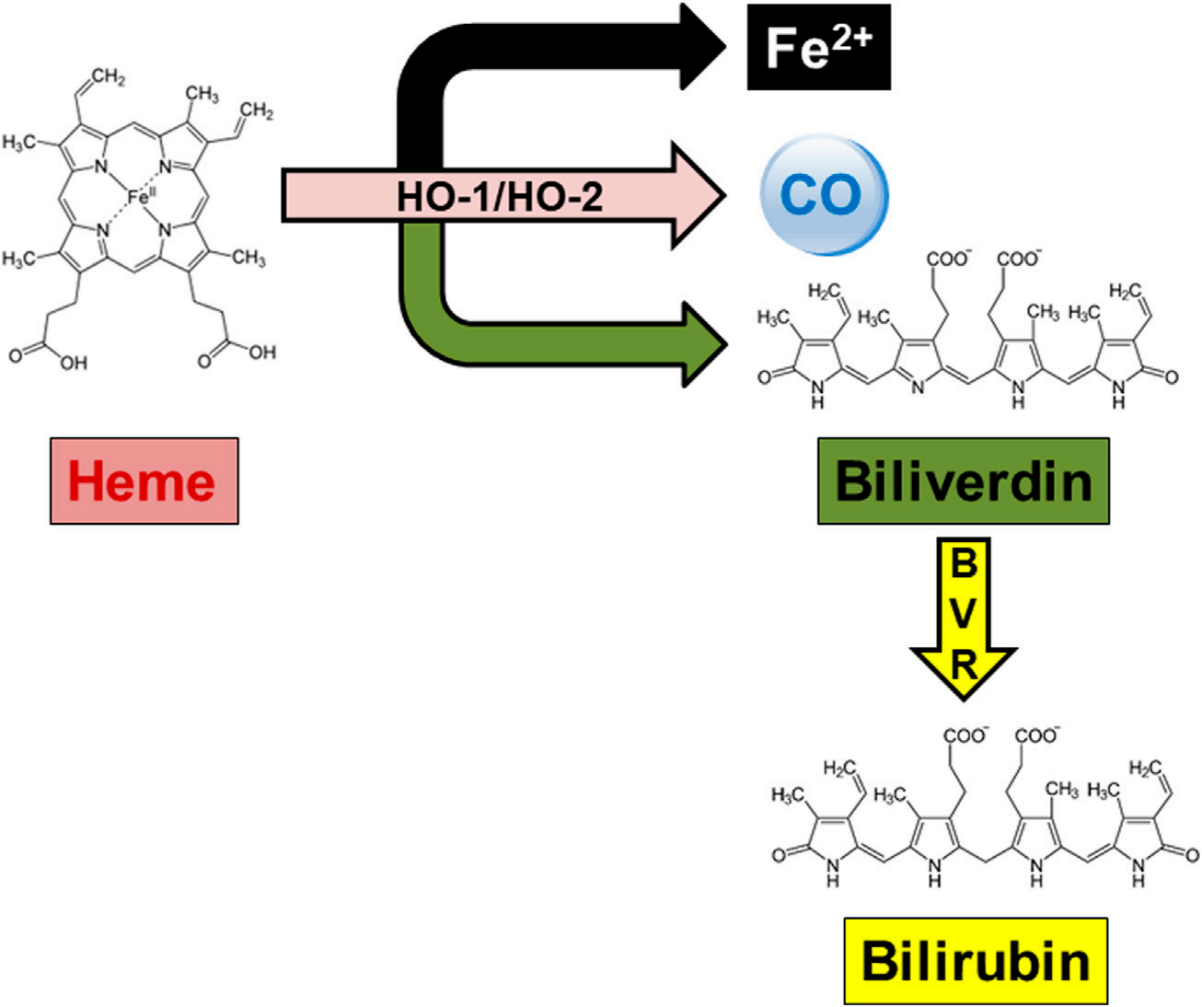
The heme metabolic pathway. In the presence of oxygen and reducing equivalents provided by the NADPH-cytochrome P-450 reductase, both heme oxygenase (HO)-1 and HO-2 oxidatively cleave the hemoproteins’ heme moieties at the α-meso-carbon bridge, resulting in equimolar amounts of ferrous iron (Fe^2+^), carbon monoxide (CO) and biliverdin. Biliverdin is further reduced at the C10 (γ bridge) by the NAD(P)H-requiring enzyme biliverdin reductase (BVR), yielding bilirubin as the final product.

Heme oxygenase (HO) is a well-known family of enzymes primarily involved in heme metabolism (Maines, 1997; Maines, 1988). To date, two HO isoforms have been described, and named HO-1 and HO-2 (Maines, 1997; Maines, 1988). A third HO isoform, HO-3, has been also described, but the strong structural similarities with HO-2 support the idea that HO-3 is a splice variant of HO-2, rather than a distinct isoform (Hayashi et al., 2004). Although HO-1 and HO-2 share the same mechanism of action, namely, the oxidation of heme, the prosthetic group of hemoproteins, in carbon monoxide (CO), ferrous iron and biliverdin (BV), these isoforms differ in regulation and distribution (Maines and Panahian, 2001) (Figure 1; Table 1). Heme oxygenase-1, the inducible isoform, is almost ubiquitous, although quite abundant in hemocatheretic organs (e.g., the liver and spleen) where aged red blood cells are destroyed and hemoglobin is recycled (Maines, 1997). Moreover, under pro-oxidant and/or pro-inflammatory conditions, HO-1 undergoes upregulation and the resulting decrease in redox-active heme and increase in antinflammatory CO both exert a strong antioxidant effect and contribute to the restoration of homeostasis (Mancuso et al., 1997a; Mancuso et al., 1997b; Maines, 1997; Motterlini and Otterbein, 2010; Ryter, 2020). Due to these cytoprotective features, HO-1 is currently considered a main player in the cell stress response and a promising drug target (Mancuso et al., 2006a; Stocker and Perrella, 2006; Motterlini and Foresti, 2014; Salerno et al., 2017; Mancuso, 2022). On the contrary, HO-2, the constitutive isoform, is quite abundant in neurons, testes and kidneys, and is involved in the physiological turnover of heme (Maines, 1997; Maines and Panahian, 2001). This early characterization was challenged, later in the years, by some evidence showing that HO-2 is also involved in the regulation of lipid metabolism and intracellular gas sensing, and can be upregulated in response to few selected stimuli (Table 1) (Raju et al., 1997; Li and Clark, 2001; Shibahara et al., 2007; Vanella et al., 2013). Importantly, HO-1 and HO-2 are not self-sufficient, but they work jointly with the cognate enzyme biliverdin reductase (BVR), which transforms HO-derived BV into bilirubin (BR) (Figure 1), a strong free radical scavenger (Stocker, 2004; Mancuso et al., 2006b; Barone et al., 2009; Mancuso et al., 2012). Along with the reductase activity, BVR is a serine/threonine/tyrosine kinase at the crossroad of many intracellular systems (Gibbs et al., 2012; Mancuso, 2017; Mancuso, 2021).

**TABLE 1:** Main features of heme oxygenase isoforms.

	Heme oxygenase-1	Heme oxygenase-2
**Molecular weight**	30–33 kDa	36 kDa
**Chromosome**	22q12	16p13.3
**Transcripts**	∼1.8 kb	∼1.3 kb and ∼1.7–1.9 kb
**Distribution**	Liver, spleen, heart, kidneys, brain (hippocampus, hypothalamus, brainstem), testes, vessels, GI tract	Brain (forebrain, hippocampus, hypothalamus, basal ganglia, cerebellum and brainstem), testes, kidneys, vessels, retina, liver, myocardium, larynx, GI tract
**Regulation**	Induced by	Constitutive, but induced by
	•hemin	•hypoxemia
	•heat shock	•adrenal glucocorticoids
	•ROS/RNS	•opioids
	•LPS	
	•proinflammatory cytokines (IL-1, TNF-α, IFN-γ)	
	•polyphenols	
	•drugs acting on the CNS (antiepileptics, antidepressants, antipsychotics)	
	•UV radiation	
	•arsenic, cadmium	

GI, gastrointestinal; IFN-γ, interferon- γ; IL, interleukin; LPS, lipopolysaccharide; RNS, reactive nitrogen species; ROS, reactive oxygen species; TNF-α, tumor necrosis-factor-α; UV, ultraviolet.

The distinctive involvement in health and diseases underlies the different appeal of HO isoforms for the scientific community. In particular, the antioxidant and cytoprotective effects of inducible HO-1 in neurodegenerative, cardiovascular and metabolic disorders, as well as in cancer, have fascinated a broad audience of investigators, resulting in more than 14,900 publications. On the other hand, the important, yet limited, physiological role has made HO-2 a niche topic, acknowledged only by a handful experts in the field.

In this context, the aim of the present article is not to provide a detailed and comparative overview of HO-1 and HO-2 features as the interested readers can refer to comprehensive reviews on this topic (Maines and Panahian, 2001; Schipper et al., 2019; Mancuso, 2022). The goal of this article is rather to shed light on HO-2. Starting from an original bibliometric analysis of the literature on HO-2, the attention will be geared towards the most significant scientific achievements which make HO-2 a potential area of successful research and an intriguing target for disease-modifying therapies.

## 2 Materials and Methods

### 2.1 Search strategy and literature inclusion

A research was conducted on 10 January 2023 and relevant studies were retrieved from the Pubmed and Scopus archives. The purpose was to include and analyze only papers focusing on HO-2, excluding those in which this latter was only one of many MeSH terms. Therefore, the strategy was to search Pubmed and Scopus for the keywords “heme oxygenase-2” or “haem oxygenase-2” only in the article title. No restrictions were imposed on publication type, year and language. Neither “Erratum” nor “Abstracts” nor “Retracted” articles were included in the analysis.

### 2.2 Data extraction and statistical analysis

The following information was extracted and analyzed for the included articles: publication type, journal, title, authors, publication year, language, citation count, subject area, Country, Institution and keywords. Microsoft Excel 2013 and Graphpad Prism 8.0.2. Were used for the analysis and graph drawing.

## 3 Results

**FIGURE 2:**
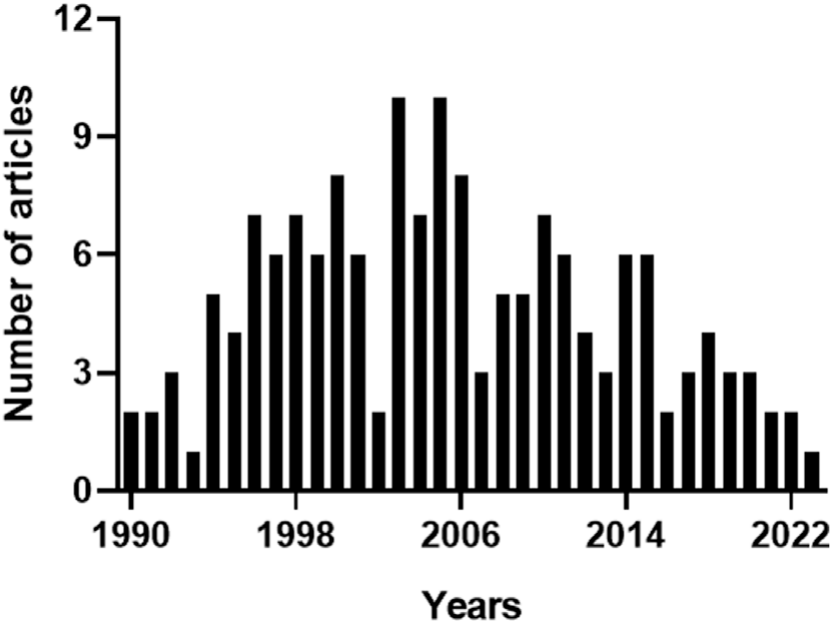
Annual number of articles on heme oxygenase-2. The 159 articles containing the keywords “heme oxygenase-2” or “haem oxygenase-2” in the title, and matching both Pubmed and Scopus archives, were sorted by publication year. 2003 and 2005 were the years with the highest number of articles published. For further information, see text.

In the Pubmed, the query “heme oxygenase-2 OR haem oxygenase-2”, with the keywords typed in the title field, found 161 articles. Since the same query generated 175 results in Scopus, due to the different coverage of literature, the bibliometric analysis included only those papers found in both the databases to standardize the results as described in the Materials and Methods section. The articles retrieved were 159, including 4 conference papers, 2 reviews and 1 editorial. Figure 2 shows the annual number of publications, and highlights that 2003 and 2005 were the years with the highest number of articles published, most of them dealing with the neuroprotective (Chang et al., 2003; Goto et al., 2003; Chen J. et al., 2005; Chen Z. et al., 2005; Namiranian et al., 2005) or cardioprotective effects of HO-2 and its by-product CO (Gagov et al., 2003; Govindaraju et al., 2005; Ishikawa et al., 2005). Table 2 describes the distribution of total publications for subject area. Almost all the articles were written in English, only 2 in Russian and 1 in Chinese.

**TABLE 2:** Subject areas of the heme oxygenase-2-related publications published since 1990.

Subject area	Number of publications^a^
Biochemistry, Genetics and Molecular Biology	98
Medicine^b^	57
Neuroscience	40
Pharmacology, Toxicology and Pharmaceutics	11
Chemistry	7
Immunology and Microbiology	4
Health Professions	3
Agricultural and Biological Sciences	3
Chemical Engineering	2

^a^ A publication can be assigned to more than one subject area.

^b^ This subject area mainly includes papers dealing with the cardiovascular, neurological and renal aspects of heme oxygenase-2.

**FIGURE 3:**
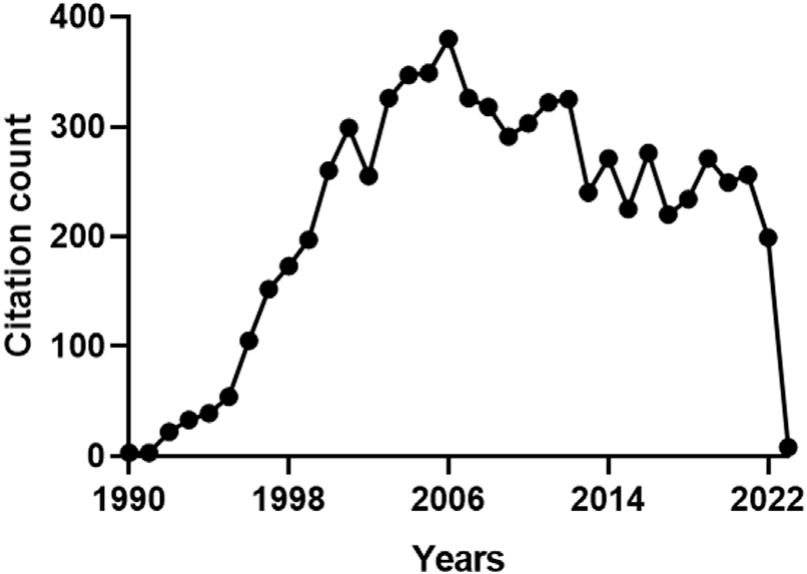
Annual number of citations received by the publications on heme oxygenase-2 (HO-2). The total citation count received by the 159 papers focusing on HO-2 was 7331. A multimodal pattern of distribution over the years can be identified: an early phase (1990–1994) characterized by a low count (100 citations), followed by a sharp increase over the next 11 years (2517 citations), a peak in 2006 and a progressive decrease, starting in 2007 and still persisting. For further information, see text.

With regard to the citations received by the 159 articles on HO-2, the total count was 7331 and the average number of citations per paper was 46 (max. 618, min. 0). Figure 3 shows that the citations followed a multimodal pattern of distribution over the years, characterized by an early phase (1990–1994) with a rather low count (100 citations), followed by a sharp increase over the next 11 years (2517 citations). A possible explanation for this thriving of citations, peaking in 2006, is that the articles published during the period 1990–1994 explored basic issues related to HO-2 (e.g., structure-activity relationship, regulation or distribution) (Ewing and Maines, 1991; Ewing and Maines, 1992; McCoubrey and Maines, 1994; Weber et al., 1994) and, therefore, were appealing only for few committed scientists. During the following 11 years, instead, HO-2 drew the attention of a broader audience of researchers (including neurologists, cardiologists, pulmonologists and gastroenterologists), who had been fascinated by a significant number of papers reporting the biological effects of HO-2 in organs and tissues (Zakhary et al., 1996; Canning and Fischer, 1998; Miller et al., 1998; Doré et al., 1999a; Doré et al., 1999b; Cao et al., 2000). The inevitable decline, which began in 2007 and still persists, reflects a lack of interest in HO-2 due to the overwhelming attention captured by HO-1, whose involvement in several cytoprotective functions, including the adaptive stress response, mesmerized the scientific community (Calabrese et al., 2010; Catino et al., 2016; Mhillaj et al., 2018). For instance, free radical overproduction is the common denominator of many diseases, such as nephropathies, and HO-1 upregulation in tubules and glomeruli counteracts oxidative damage and helps in preventing the progression of renal dysfunction into end-stage renal disease (Wiesel et al., 2001; Chung and Perrella, 2004; Lever et al., 2016). The evidence that HO-1 knockout mouse exhibits exacerbation of chronic renovascular hypertension and acute renal failure, further confirms the importance of this isoform in renal pathophysiology (Wiesel et al., 2001). These findings mutually support the hypothesis that HO-2, despite being quite abundant in the kidney, is unable to compensate for the loss of HO-1 and contrast the progressive worsening of renal function. A reasonable explanation for this failure is the inability of HO-2 to undergo upregulation in response to oxidative stress and prevent kidney injury.

The analysis of the number of published papers along with the citations received, allows tracing the research groups who mainly contributed to the advancement of this field. Mahin Maines and her Fellows, at the University of Rochester, paved the way in 1990, providing dozens of milestone articles covering basic and applied aspects of HO-2. Shigeki Shibahara and Collaborators, at the Yamagata University and then at the Tohoku University, focused on the hypoxia-related regulation of HO-2 since 1995 and were the first to propose the novel role of this protein as an oxygen sensor in 2006. Since 1996, at the Johns Hopkins University, Solomon H. Snyder and his research group have provided novel insights about the neuroprotective effects of HO-2 and its by-product CO. Together with his former Fellow, Sylvain Dorè, Solomon H. Snyder also highlighted the neuroprotective effects of HO-2-derived BR. At the Thomas Jefferson University, Raymond F. Regan and his group have focused the attention on the neuroprotective *versus* neurotoxic effects of HO-2 since 2003. In 2007 Stephen W. Ragsdale’s research group, at the University of Nebraska, ideally passed the relay, by working on the heme-HO-2 interaction and the transcription factor-based activation of this enzyme. The Ragsdale group published the last paper on HO-2 a few months ago. Interestingly, among the 10 most-cited articles (Table 3), 4 papers were authored by Mahin D. Maines’ and 3 by Solomon H. Snyder’s laboratories.

**TABLE 3:** The top 10 most-cited articles on heme oxygenase-2.

Title	Senior author	Affiliation	Citations
Bilirubin, formed by activation of heme oxygenase-2, protects neurons against oxidative stress injury	Solomon H. Snyder	The Johns Hopkins University School of Medicine	618
Heme oxygenase 2: endothelial and neuronal localization and role in endothelium-dependent relaxation	Solomon H. Snyder	The Johns Hopkins University School of Medicine	357
Rapid induction ofheme oxygenase 1 mRNA and protein by hyperthermia in rat brain: Heme oxygenase 2is not a heat shock protein	Mahin D. Maines	University of Rochester Medical Center	250
Targeted gene deletion of heme oxygenase 2 reveals neural role for carbon monoxide	Solomon H. Snyder	The Johns Hopkins University School of Medicine	213
*In situ* hybridization and immunohistochemical localization of heme oxygenase-2 mRNA and protein in normal rat brain: Differential distribution of isozyme 1 and 2	Mahin D. Maines	University of Rochester Medical Center	187
Oxygen toxicity and iron accumulation in the lungs of mice lacking heme oxygenase-2	Kenneth D. Poss	Stanford University School of Medicine	183
Characterization of rat heme oxygenase-3 gene. Implication of processed pseudogenes derived from heme oxygenase-2 gene	Masato Noguchi	Kurume University School of Medicine	179
Human heme oxygenase-2: characterization and expression of a full-length cDNA and evidence suggesting that the two HO-2 transcripts may differ by choice of polyadenylation signal	Mahin D. Maines	University of Rochester Medical Center	173
Heme oxygenase-2 is a hemoprotein and binds heme through heme regulatory motifs that are not involved in heme catalysis	Mahin D. Maines	University of Rochester Medical Center	162
Hippocampal long-term potentiation is normal in heme oxygenase-2 mutant mice	Susumu Tonegawa	Howard Hughes Medical Institute, Massachusetts Institute of Technology	155

## 4 Discussion

Over the years, HO-2 has received far less attention from the scientific community than HO-1. The reasons for this underestimation can be traced back to the HO-2 constitutive activity and poor inducibility (Table 1). These features reduced researchers’ appreciation for HO-2, in particular clinicians, who felt the impossibility to explore the applied outcomes of HO-2 upregulation, as an insurmountable hurdle. In light of this, it is not surprising that most of the article published since 1990 falls in the basic subject area of Biochemistry, Genetics and Molecular Biology (Table 2). This trend is confirmed when considering the 10 most-cited articles, 6 of which address the activity, regulation and localization of HO-2 (Table 3). Among the articles listed in Table 3 is the one by Susumu Tonegawa’s group describing the HO-2 null mouse, an experimental model used worldwide to investigate the many roles of the constitutive isoform and its by-products (Poss et al., 1995; Zakhary et al., 1997; Doré et al., 1999a). This transgenic mouse exhibits a significant downregulation of HO-2 gene expression in target organs, such as the brain and testes, but it is fertile and maintains physiological grooming and feeding behaviors (Poss et al., 1995). Moreover, neither the brain nor the peripheral organs of the HO-2 null mouse display significant histological modifications with respect to the wild-type animal (Poss et al., 1995). Later studies have expanded the knowledge about the HO-2 null phenotype and unravelled the involvement of this isoform in both the non-adrenergic-non cholinergic relaxation of the gut and antinflammatory response and wound healing and endothelial cell function (Zakhary et al., 1997; Bellner et al., 2015; 2009; Halilovic et al., 2011). As far as I know, HO-2 deficiency has not been described in humans, yet.

The role of HO-2 as a drug target has also been neglected: just to clarify, only 11 publications fall in the subject area of Pharmacology, Toxicology and Pharmaceutics (Table 2). In this regard, only a couple of articles have explored the link between HO-2 and drugs, namely, those by Chen Z. et al. (2005) and Li and Clark (2001), which described the involvement of rat HO-2 in either the neuroprotective effects of quetiapine and venlafaxine or the morphine-induced tolerance/withdrawal phenomena. However, a careful analysis of the literature published after 2005 allows gathering significant pieces of evidence supporting the role of HO-2 as drug target. In 2011–2013, Kanji Nakatsu and co-workers reported that menadione and clemizole, two well-known drugs, displayed agonist and antagonist activities on HO-2, respectively (Vukomanovic et al., 2011; Vlahakis et al., 2013). These findings were confirmed and extended by other scientists who synthesized and tested novel menadione- and clemizole-derived agonists and antagonists, with improved potency/selectivity on HO-2 (Kong et al., 2015; Salerno et al., 2015; Intagliata et al., 2019; Floresta et al., 2020). Unfortunately, the availability of both HO-2 activators and inhibitors did not encourage scientists to perform *ad hoc* studies to address the effects of these pharmacological tools in animal models of diseases, and these interesting lines of research, worthy of further study, remained almost unknown (the citations received by the related papers are in the range 4–52). On the contrary, it is my opinion that these activators/inhibitors would be very useful to clarify some controversial aspects of HO-2, such as those highlighted below.

The role of HO-2 in cerebrovascular diseases is still unclear, because some evidence suggests that the enzyme is neuroprotective, whereas other studies support the opposite hypothesis (Doré et al., 1999a; Doré et al., 2000; Goto et al., 2003; Qu et al., 2007; Chen-Roetling et al., 2014; Goto et al., 2018). A possible explanation for this apparent conundrum is the experimental protocol used. The studies suggesting the neurotoxic effects of HO-2 were carried out in HO-2 null mice whose brains were removed 15–17 days *postpartum*, cortical neurons isolated and cultured for 12–16 days *in vitro* and then exposed to exogenous blood or hemin to mimic the hemorrhagic injury. Conversely, the articles dealing with the neuroprotective effects of HO-2 were performed in adult HO-2 null mice, exposed to middle-cerebral artery occlusion (1 h occlusion followed by 23 h reperfusion) or collagenase injection, to mimic ischemia or hemorrhage, respectively, and whose brains were removed and analyzed just after the end of the experiments without any further *in vitro* manipulation. Although it is necessary to use a cautious approach, it is still essential to consider that these divergent results may also depend on the animal model itself. Indeed, the products of many genes are essential for normal function, and inactivating these genes may induce age-dependent, morphological or physiological abnormalities (Nelson, 1997). The lack of HO-2, the main isozyme in the central nervous system involved in the synthesis of neuroprotective CO and BR, could have impaired the brain response to injury. The young age of the HO-2 knockout mice whose brains were processed to prepare neuronal cultures, and the long incubation of dispersed neurons before *in vitro* treatments, could have contributed to exacerbate the toxicity of exogenous blood or hemin. The possible age-dependent compensative overexpression of antioxidant genes, including HO-1, occurring in adult HO-2 null mice exposed to either *in vitro* or *in vivo* ischemia/hemorrhage, is a confounding factor not completely resolved, yet. The availability of HO-2 inhibitors could help to design comparable studies in adult mice with normally developed brains, without any compensative gene overexpression, providing researchers the possibility to confirm previous results or propose novel hypotheses. The possibility to modulate HO-2 in another rodent species frequently used to induce cerebral ischemia or hemorrhage, such as the rat, is another advantage provided by the pharmacological tools and should be taken into proper consideration.

Interestingly, HO-2 could be a new target for pharmacogenetic studies. The HO-2 genetic variants *rs2270363* and *rs1051308* (−42 + 1444A>G-HMOX2 and c.544G>A polymorphisms, respectively) have been linked to Parkinson’s disease (PD). The homozygous G/G genotype of both the *rs2270363* and *rs1051308* variants was associated with the risk of developing PD in Spanish and Han Chinese subjects, respectively (Ayuso et al., 2011; Tian et al., 2017). Noteworthy, the GG genotype of *rs1051308* variant increases the risk of PD in Han Chinese men, but not in women (Tian et al., 2017). The relevance of these important studies (gone almost unnoticed, as attested by the citation count of 3 and 16 for the most recent and the oldest article, respectively) could be even greater considering that they fall into two hot areas of interest, such as geriatrics and gender medicine.

In conclusion, the purpose of this article will be achieved if the readers, in particular young scientists not yet committed to a specific area of research, get passionate about HO-2 and its unexplored potential as drug target. The availability of selective HO-2 agonists and antagonists is indeed a plus to take advantage of. At the meantime, this paper will have hit the mark if researchers give the right credit to bibliometric indexes, considering the number of citations of an article as a misleading index, at times. The history of HO-2 should be taken as an example. Indeed, as discussed in this article, ideas for new discoveries can also be found in apparently niche topics, as long as the scientist is fully aware of the research field and its translational potential.

## Data Availability

Publicly available datasets were analyzed in this study. This data can be found here: www.pubmed.ncbi.nlm.nih.gov www.scopus.com.
